# Mechanism for large-scale canyon deformations due to filling of large reservoir of hydropower project

**DOI:** 10.1038/s41598-020-69167-9

**Published:** 2020-07-22

**Authors:** Hui Jiang, Chu-Han Zhang, Yuan-De Zhou, Jian-Wen Pan, Jin-Ting Wang, Ming-Xin Wu, Qi-Xiang Fan

**Affiliations:** 1 State Key Laboratory of Hydroscience and Engineering, Tsinghua University, Beijing, 100084 China; 2 China Renewable Energy Engineering Institute, Beijing, 100120 China; 3 China Three Gorges Corporation, Beijing, 100038 China; 4 Present Address: China Huaneng Group CO., LTD, Beijing, 100031 China

**Keywords:** Hydrology, Civil engineering

## Abstract

Large storage dam projects may modify geo-environmental conditions in many ways. The reservoir impoundment of the 285.5 m high Xiluodu arch dam located on the Jinsha River (China) caused large-scale canyon deformations, including significant canyon contraction and uplift movements from reservoir to downstream valley. The dam experienced subsequent tilting towards upstream and raised a safety concern of the project. A Thermo-Hydro-Mechanical (THM) mechanism is proposed for this extraordinary behavior. Due to reservoir impounding and seepage, significant temperature drops and fluid pressure increase within the underlying geothermal limestone aquifer in a synclinal basin are primary root causes. Finite element THM simulations successfully reproduce these unique deformations. Recent observations of large quantities of thermalized discharge water downstream and high pore pressure in the limestone layer provide further support for the proposed mechanism. Furthermore, refined numerical modeling is adopted to evaluate the safety of Xiluodu dam subjected to potential larger canyon contractions. We conclude that these unprecedented phenomena are dominantly the consequence of THM response to regional hydrogeological evolution following the build-up of a large reservoir. The accumulated canyon contractions at the current stage would not pose a direct threat to the dam safety, but a tripled situation may cause severe safety issues.

## Introduction

A number of large hydropower stations have been built in Southwest China during the past decades, including several dams up to 300 m high together with huge reservoirs storing billion tons of water. The development of such hydropower projects provides flood storage space, clean electricity, and irrigation water. At the meantime, environmental impacts of these large structures have attracted intense public attention and led to more in-depth research and policy assessments^1–4^, particularly because most of these projects are located around the edge of the Tibet Plateau, known as ‘The Water Tower of Asia’, one of the Earth’s most sensitive environments.

Canyon deformation is regarded as an important geo-environmental impact of hydropower projects. The construction of the Xiluodu arch dam, a large hydropower station located on Jinsha River, introduced substantial canyon deformations with unique characteristics. There were precedents in dam developments that presented contractive canyon deformations along the transverse river direction. For instance, Zeuzier arch dam^5^ in Switzerland and Beauregard arch dam^6,7^ in Italy suffered from severe cracking due to canyon deformations, and the latter was partially demolished to lower the reservoir elevation by about 52 m (half of the total water head) for safety. In China, field monitoring data from Jinping arch dam (the world’s highest dam at 305 m)^8,9^, and Lijiaxia arch dam^8^, also presented a contractive deformation up to 35 mm at canyons following reservoir impoundment. It should be emphasized that the valley deformations at these dam sites occurred in a local manner. Multiple mechanisms have been proposed for explaining the above canyon contractions^5,6,9–11^, amongst which saturation-induced strength degradation, damage and creeping of rocks within the valley slopes saturated or semi-saturated by reservoir water have been widely accepted as major mechanisms. However, the relevant mechanisms proposed in previous studies, including the ancient landslide reactivation, saturation-induced rock strength degradation, reservoir loads, and excavation induced unloading, are all incapable of providing a comprehensive explanation for the large-scale canyon deformations and uplifts at the base monitored in Xiluodu dam site. Jiang^12^ and Yin et al.^13^ both emphasized the significance of incorporating the geo-thermal role in the canyon deformation analysis. Their reports show that an individual factor such as the interlayer slippage of abutments or the variation in geo-thermal field cannot account for the unprecedented phenomena reasonably. Based on long-term and comprehensive monitoring data of temperature, seepage pressure and rates, as well as deformations in the site, a Thermo-Hydro-Mechanical (THM) mechanism is proposed in this study. The performance of the corresponding coupled numerical model is demonstrated through a thorough comparison between simulation results and all relevant field data, and the capability of the proposed mechanism of reproducing large-scale canyon deformations is validated. Moreover, extension analyses are conducted to quantify the contributions of the most important influential factors, and to evaluate the dam safety due to canyon deformations.

## Field observation of canyon deformations

Xiluodu arch dam has a reservoir storage of 12.67 × 10^9^ m^3^. With an installed capacity of 13,860 MW, its power station is one of the largest on the Yangtze River. From the onset of reservoir impoundment in May 2013, large-scale canyon deformations were observed, featuring a uniform contraction along the transverse river direction, and uplifts both at the dam base and over several kilometers of the downstream valley floor. This unusual behavior of valley deformation had brought forward a wide range of spirited discussion on its root-cause mechanism, which gave an impetus for the current investigation.

**Figure 1:**
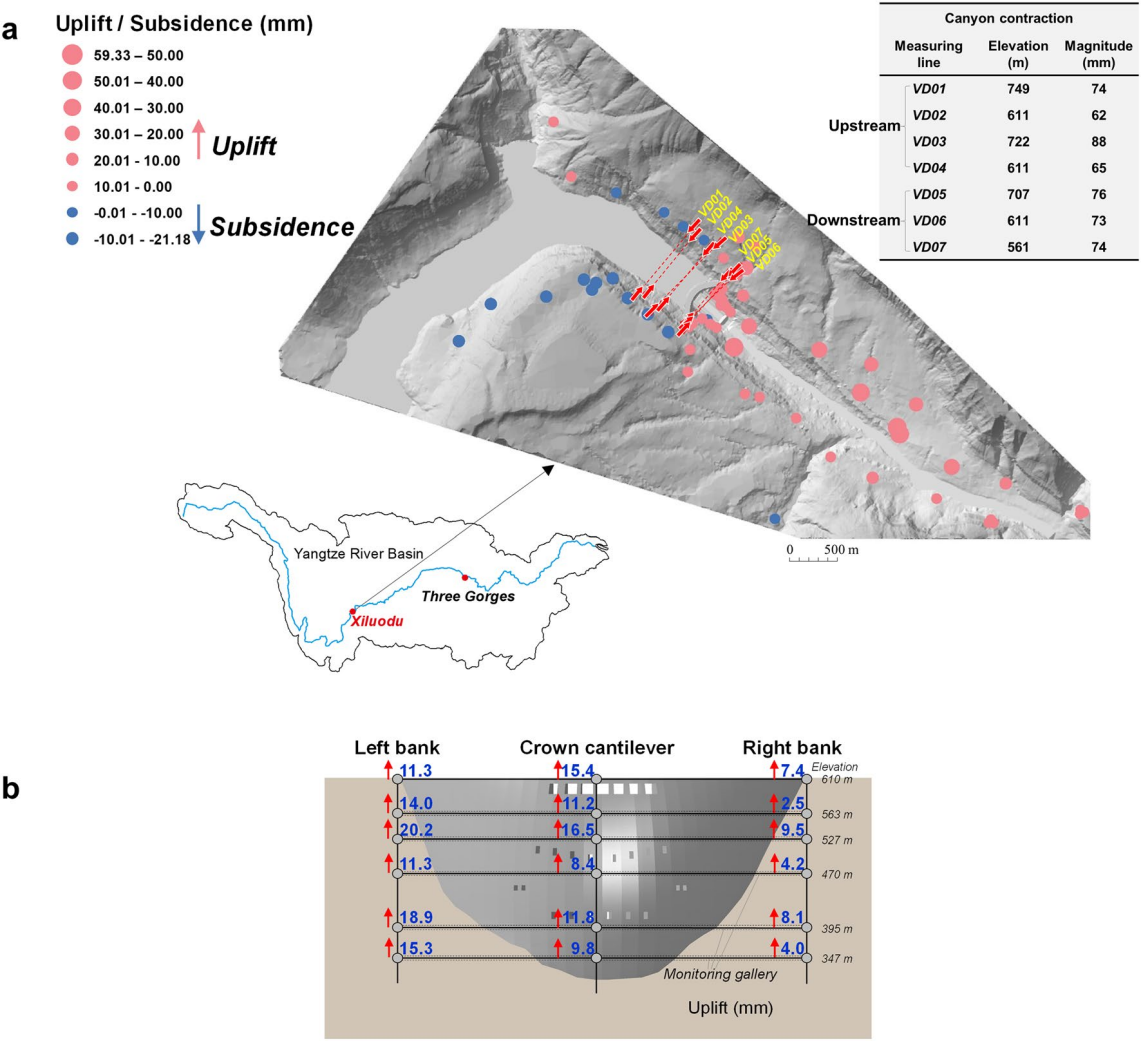
A scenario of canyon deformations (Jan 2018). (**a**) Canyon contraction, reservoir subsidence and downstream valley uplift. Seven canyon contraction monitoring lines are highlighted in red, covering a 700-m-long and 200-m-high range from reservoir banks to downstream valleys. Spatial distribution of vertical displacements from upstream to downstream bedrocks are shown as colored circles, among which uplift is indicated by red and subsidence by blue (the downstream subsidence by water load at the early impounding stage subtracted). (**b**) Maximum uplift at the dam base after impoundment, presenting an overall oblique movement. The values on right bank are significantly smaller due to the reservoir geography curved to the right bank (see (**a**)).

Based on geodetic monitoring data, canyon contraction of 40–50 mm was triggered at the early filling stage, which increased to 62–88 mm at the end of the fourth storage cycle (Fig. 1a, Supplementary Fig. S1). After a monotonic increase over 5 years, the contraction rate slowed down and currently remains at 3–7 mm per year. Furthermore, field measurements have shown normal, accumulative settlements at the reservoir bottom, but an abnormal deformation of uplifts was observed at the dam base as well as at downstream valley floor (Fig. 1a,b, Supplementary Fig. S2). Maximum uplifts at the left and right sides of the dam base are 11.3–20.2 mm and 2.5–9.5 mm, respectively, and reach a peak uplift of 60 mm at the downstream bedrocks, presenting an overall trend of greater upward movements at the left bank. This observation is different from the common pattern of valley deformation at most dam sites, that is, the settlement at the reservoir bottom causing associated vertical subsidence at dam base and downstream bedrocks, as evidenced by the monitoring data of Xiaowan arch dam^8^, Hoover arch dam^14^ and Longyangxia arch dam^8^. The extraordinary canyon deformation pattern at the site of Xiluodu dam also led to abnormal displacement mode of the dam structure. Due to the canyon contraction and dam base—downstream valley uplift, an accumulative horizontal displacement of 52 mm was observed at the dam crest towards the upstream direction after four cycles of reservoir storage, which is opposite to the general pattern of downward displacement by water pressure loads.

## Results and discussions

### Geological setting and hydrogeological investigations

**Figure 2:**
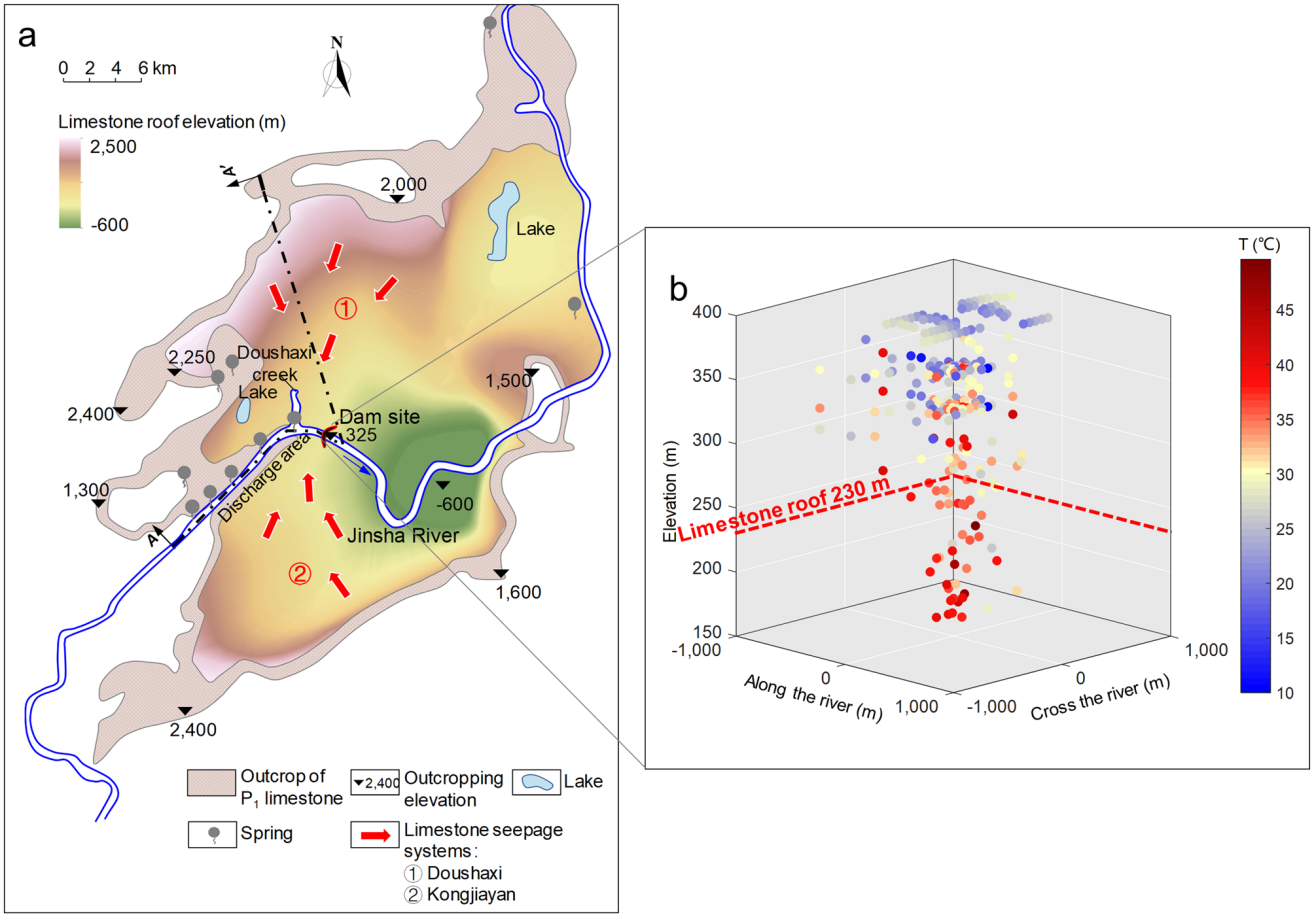
Overview of hydrogeological conditions. (**a**) Hydrogeological characteristics of P_1_ limestone layers (the overlying basalt layers are removed for clarity). Jinsha River deep cut across the basin. The P_1_ limestone layer is exposed along the perimeter of the basin, with an outcropping elevation of 1,000–2,000 m, and the strong development of karst promotes its permeability, resulting in the formation of two seepage systems in the vicinity of the dam site: the Doushaxi system at the Northwest and the Kongjiayan system at the Southwest. (**b**) Geothermal characteristics within the foundation rock. Measuring points of high temperature are indicated by red circles.

**Figure 3:**
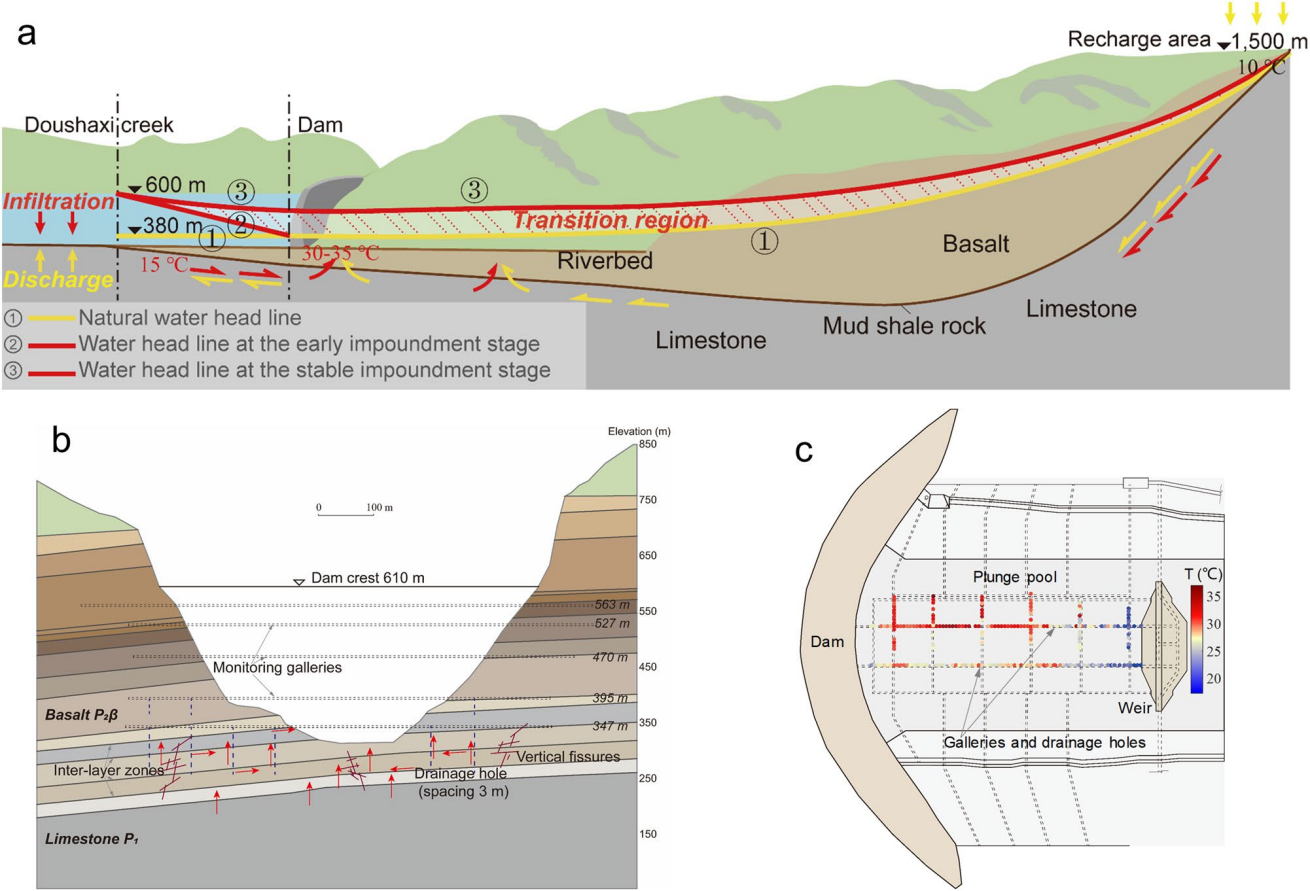
Schematic diagram of hydrogeological changes due to reservoir impounding. (**a**) Section A-A′ in Fig. 2a. Lines ①–③: Water levels of the limestone at different stages. Line ①: The natural water level prior to reservoir impoundment, seepage path is indicated by yellow arrows. Line ②: The water level from Doushaxi creek to the dam site at the early filling stage, resulting in cool water (15 °C) infiltrating into geothermal limestone layers (red arrows). Line ③: The steady water level for reservoir normal operations. (**b**) A schematic of seepage paths in the foundation layers along the cross-river section. (**c**) The monitoring results of discharge water temperature at the drainage holes of the plunge pool. Red circles indicate a high temperature up to 30–35 °C.

Xiluodu dam site is located in the Yongsheng synclinal west wing of the Leibo-Yongshan tectonic rhombic basin with axis dimensions of 35 km and 25 km in NE and NW directions, respectively (Fig. 2a). The base rock beneath the dam is composed of layered basalts dipping towards the SE, i.e., the downstream left bank direction, at an angle lower than 20°. The rock mass at both river banks is mainly composed of the Permian upper series basalt P_2_β. The underlying lower series limestone P_1_, 400–500 m in thickness, is exposed along Doushaxi creek at 2–3 km upstream, and deeply buried under a 90 m thick basalt layer at the dam site (Fig. 3). A 2–3 m thick layer of mud shale rock embeds along the interface with the fractured basalt layer, and serves as a water insulation layer owing to its lower permeability. However, the partial absence of this mud shale layer discovered by geological survey allows for hydraulic communication between the limestone and fractured basalt through seepage networks. Two seepage systems (Fig. 2a) are observed during a site survey for the P_1_ limestone layer, which are recharged from high mountain areas of the basin, and discharged in the exposure zone upstream of the dam site, as a series of springs along the river banks. Moreover, the burial depth of the limestone layer downstream increases with the distance from the dam site.

Based on geological data, two hydrogeological characteristics of the P_1_ limestone layer underneath the dam site can be established: (i) a pressure-bearing zone with a water head 1.5–2.5 m higher than the river water level due to seepage from recharge areas; (ii) significantly elevated ground temperature (Fig. 2b) increasing with depth in a range of 33–49 °C (38.8 °C in average). Both characteristics are natural outcomes of the geological basin setting as explained in Fig. 3. The groundwater recharge of the P_1_ limestone aquifer mainly comes from atmospheric precipitation, surrounding the basin with an average recharge elevation of 1,000–2,000 m and an annual average temperature of 10 °C. In-situ measurements of elevated temperature within the limestone aquifer reflect the effect of the geothermal gradient of about 3–6 °C /100 m from the recharge zone downwards to the limestone layer beneath the dam base with elevation of 235 m.

### THM mechanism for canyon deformations

Above hydrogeological characteristics before reservoir impoundment provide the foundation of a THM interaction mechanism that we propose to account for the canyon deformations. Figure 3a is based on a hydrogeological survey for water head in limestone layers before and after impounding. Line ① (Fig. 3a) denotes the pre-impounding natural water head with a hydraulic gradient of 1/300 near the dam site. Following reservoir buildup, elevated pressure gradient led to infiltration from the reservoir into the limestone aquifer (Line ② and red arrows in Fig. 3a). Furthermore, the seepage network within the basalt layers, including high permeable inter-layer zones, vertical fissures, and drainage holes, allowed a subsequent cross-zone seepage flow (Fig. 3b). As the water temperature of reservoir seepage (15 °C in average) is significantly lower than the ambient temperature of the host rocks, a cooling effect occurred and triggered a thermal contraction in the limestone layer. These THM coupling effects under large pressure gradient were responsible for the temperature drop within the dam foundation during the early impounding stage of May 2013 to May 2015, and the resulting canyon contraction (about 70–80% of the total magnitude observed). Meanwhile, the increase in seepage water pressure during reservoir build-up drove the uplift of dam foundation and downstream valleys.

After several cycles of water impoundment, hydraulic gradients and flow rates for reservoir water into the limestone aquifer and cross-zone discharge should be approaching a steady state (Line ③ in Fig. 3a). Hence the canyon deformations can be expected to become gradually stable when the deep hydro-thermal circulation reaches a new status of balance.

Monitoring results of water temperature and seepage flow at the bottom of the plunge pool during the impounding process provided further support for the above mechanism (Fig. 3c). A significant temperature rise was observed in the plunge pool discharge from the initial 15 °C of infiltrated reservoir water to a higher value of 30–35 °C. The heat source of the plunge water can only come from the cross-zone seepage flow through the deeply buried limestone layer, which then percolated through the basalt fissures and into the drainage system of the plunge pool.

**Figure 4:**
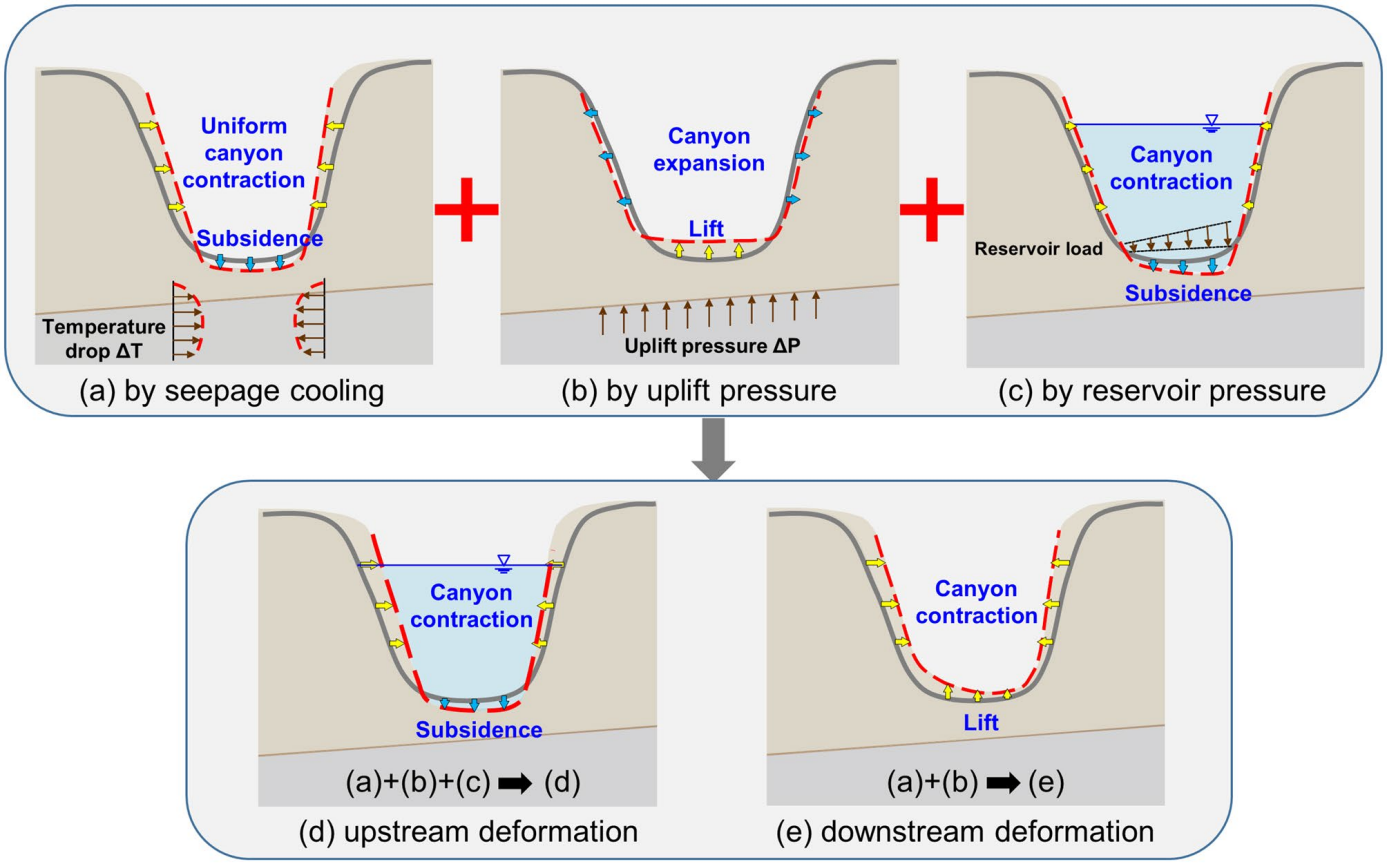
Geomechanical scenario of canyon deformations. (**a**) The cooling of foundation rock makes a major contribution to the uniform canyon contraction and a minor effect on the subsidence of valley bedrocks. (**b**) Seepage uplift pressure on the limestone roof mainly causes an uplift of dam base-valley rocks and a minor effect on the increase of canyon span. (**c**) The reservoir water load mainly contributes to the subsidence of the reservoir bottom, and can also slightly cause a certain magnitude of valley contraction. Considering the reservoir geometry is curved to the right bank, greater subsidence shall be mobilized on the right side. (**d**) Canyon deformations at the upstream. (**e**) Canyon deformations at the downstream.

Through decomposition of factors influencing the hydrogeological system, Fig. 4 illustrates the canyon deformation scenarios due to temperature drop (Fig. 4a), uplift pressure (Fig. 4b) and reservoir water load (Fig. 4c). For the upstream reservoir area (Fig. 4d), the above three factors all work and cause canyon contraction and subsidence of the reservoir bottom. On the other hand, for the dam base and downstream bedrocks (Fig. 4e), only the first two factors (Fig. 4a,b) take effect, which mobilize canyon contraction and uplift of foundation rocks. We may find that the patterns of superimposed deformation in Fig. 4d,e are in general accordance with the monitoring scenarios shown in Fig. 1.

### Verification and discussions

**Figure 5:**
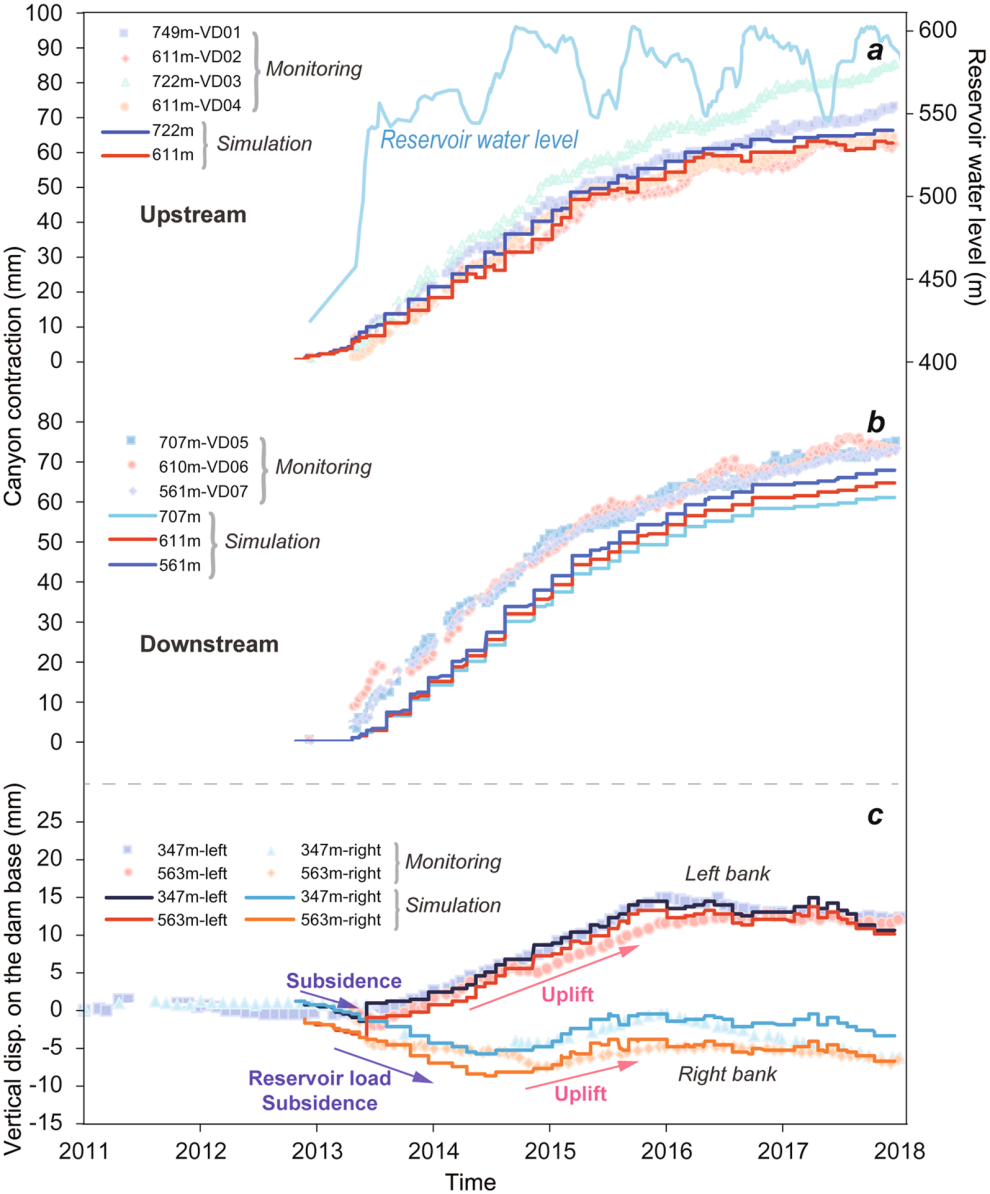
Comparison of simulation results and field measurements. Plots (**a**,**b**) present canyon contraction at the upstream and downstream, respectively. Plots (**c**) illustrate vertical deformation at the dam base.

In order to validate the proposed THM mechanism, a large-scale finite element model was developed to investigate the deformation characteristics. A statistical optimization method was adopted to obtain the spatial and temporal distributions of temperature drop and seepage uplift pressure loadings (see “Methods” section). A 30 km along river-transverse profile at the dam site was discretized. The topography was simulated and all parameters of geological rock strata were determined by in-situ exploration tests. As shown in Fig. 5, numerical results of the spatial and temporal evolution of canyon deformations are in good agreement with field measurements. Such comprehensive verification can only be achieved when the cooling effect in the underlying bedrocks, the extra seepage pressure and the difference of reservoir water load on the left and right banks are all incorporated in the model. Through decomposition analysis of each load factor, we found that the cooling of the bedrocks makes the most significant contribution to canyon contraction, and the seepage pressure mainly accounts for the uplift movements at the dam base and the downstream valleys (Supplementary Fig. S3).

Based on field monitoring, the seepage flow rate at the plunge pool reached 12 m^3^/min and stabilized at about 5–6 m^3^/min after five cycles of reservoir impoundment (Supplementary Fig. S4). As the basalt layer at the dam base has been water proofed with a complete grouting curtain, the water leakage mainly comes from the underlying limestone aquifer. A heat balance calculation was conducted based on the total seepage amount of 20 million m^3^ at the plunge pool over the previous 5 years. Consider a rise of seepage water temperature by 15–20 °C per field measurements, the heat absorbed is roughly equivalent to the thermal energy released from cooling down 80 million m^3^ of host rock by about 7.5 °C (see “Methods” section).

An ongoing drilling program started in November 2018 included 8 deep holes drilled into the limestone aquifer and 9 holes into the basalt layers. An average temperature drop of about 5–9 °C occurred within the limestone aquifer during the past six years, which is close to the aforementioned estimated value of 7.5 °C (Supplementary Fig. S5). The uplift pressures on the limestone roof reached 97–208 m water head above the corresponding downstream river level, much higher than that of 1.5–2.5 m before reservoir impounding. Moreover, most boreholes observed abrupt temperature jumps at the interlayer shear zones of basalt (Supplementary Fig. S6), and a large quantity of seepage water flow when drilling through limestone layers (Supplementary Fig. S7), both implying remarkable upflow seepage water with high temperature.

The above model-data comparisons clearly demonstrate that the proposed model can accurately represent the actual THM performance at the dam site. Furthermore, its advantages over other models or mechanisms in literature are illustrated through an inter-model comparison as follows. It is straightforward to attribute the progressive movements of abutment rocks to the accumulation of internal creep strains under seepage loads after reservoir impoundment, such as the work by Fan et al.^10^. However, their work only presents a preliminary investigation into the individual effects of each factor that may contribute to the canyon deformations. Even though their simulation results show that an assumed exaggerated temperature drop of 20 °C in the foundation rock would cause notable canyon contractions of 35–40 mm, the accompanying base settlements are contrary to the upward movements of the field measurements, and they turned to the rock creeping factor. Moreover, using commonly used creep parameters of the basalt layers, we found that the contribution of creep strains is relatively minor to the canyon deformations. Regarding the mechanism of saturation-induced rock strength degradation proposed by Cheng et al.^9^, their simulation results reasonably reproduced the impounding induced slope transverse displacements at the upstream valley of Jinping-I dam site. However, such a mechanism is deemed incapable to explain the uniform canyon contractions both at the upstream and downstream valleys in Xiluodu dam site when the difference in saturation effects on both sides is considered. In the recent work by Yin et al.^13^, the effects of geothermal variation on canyon contractions was emphasized, and it also pointed out that the cooling effect of foundation rocks mainly accounts for the canyon contractions. However, without identifying the effects of seepage uplift pressure on the limestone roof (Fig. 4b), their model work does not allow for a comprehensive comparison and discussion of uplift movements at the dam site. Overall, the THM model proposed in this paper integrates the advantages of the above models, and presents an unprecedented in-depth investigation into the hydrogeological characteristics of the site, and our analyses provide a comprehensive comparison of the canyon deformations with the in-situ multi-physics monitoring data.

### On dam safety evaluation

**Figure 6:**
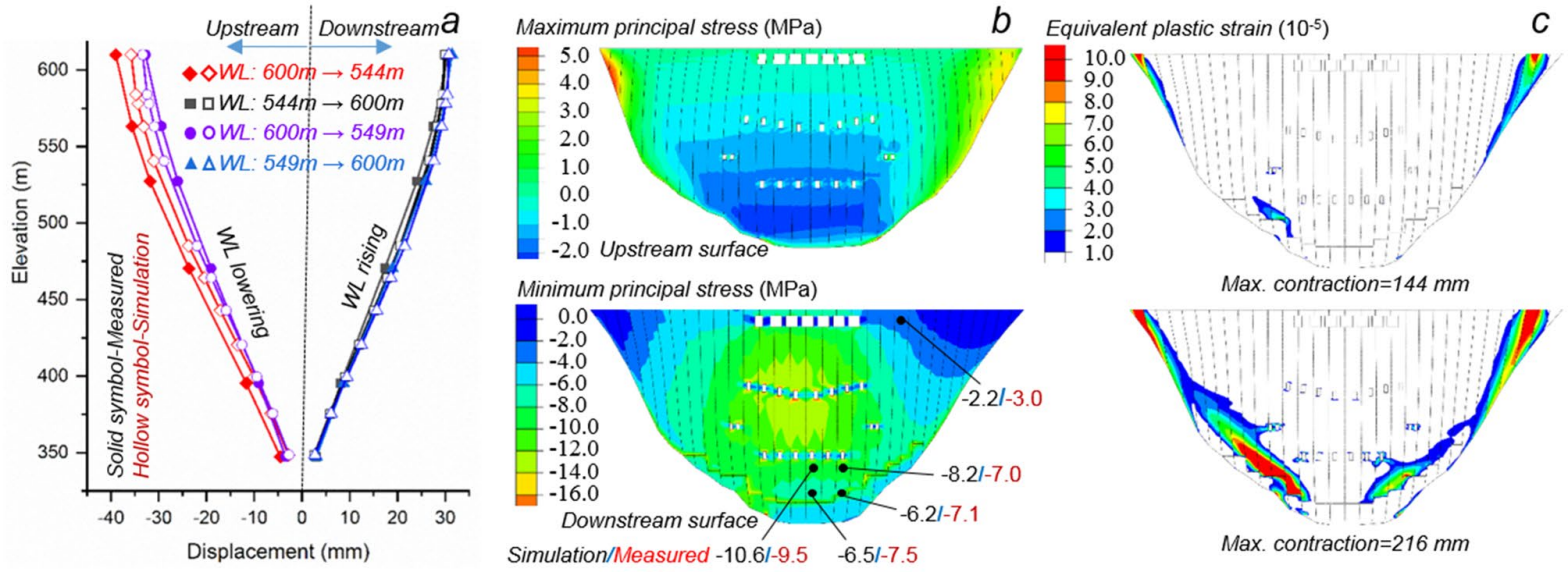
Influence of canyon contraction on dam performance. (**a**) The distribution of longitudinal displacements at Monolith 15 at the current stage (maximum canyon contraction = 72 mm) caused by the storage and drainage cycles, showing a comparison of simulation results (marked by hollow symbols) and corresponding field measurements (marked by solid symbols). (**b**) Principal stress distributions on the dam surfaces at the current stage (maximum canyon contraction = 72 mm), showing in the lower subgraph a comparison of the simulated and measured minimum components (separated by ‘/’) at typical positions on the downstream surface. (**c**) Plastic strain distributions on the downstream dam surface with the current canyon contractions doubled (upper subgraph) and tripled (lower subgraph) respectively as assumed inputs.

Reasonably the canyon deformations would interact with the dam structure and in turn impose challenges on its safe operations. A refined finite element model is established for Xiluodu arch dam, and the above back-analyzed canyon deformations are directly enforced along the dam-foundation interface. The considered load components include the structural dead-weight, hydrostatic pressure, and the temperature load. The nonlinear behavior of dam concrete was described using the elastoplastic Rankine- and Drucker-Prager criterion. The model was calibrated in terms of the longitudinal displacement at Monolith 15 (Fig. 6a) and the minimum principal stress distribution at the downstream surface (Fig. 6b). One can note that the simulation results are in good agreement with the corresponding measurements, and the bulk of dam concrete remains linear-elastic at the current stage (maximum canyon contraction = 72 mm) as evidenced by the recoverable displacements during repetitive storage-drainage cycles (Fig. 6a) and relatively low tensile and compressive principal stress distributions on the dam surfaces (Fig. 6b). However, for the two assumed cases with the current canyon contractions doubled and tripled respectively, numerical results (Fig. 6c) exhibit slight to extensive plastic strain distribution on the downstream surface by the enlargement of maximum canyon contraction from 144 to 216 mm, indicating a severe safety threat to the dam structure for the latter case.

## Conclusions

In summary, a large reservoir buildup by the construction of a hydropower project can introduce prominent changes in regional hydrogeological environmental conditions. Particularly, for a dam site in a synclinal basin with high ground temperature, water impoundment may trigger cooling effect and uplift pressures within canyon rocks, and in turn, a THM coupling mechanism may produce large-scale deformations, potentially affecting the safety of the arch dam, which is vulnerable to non-uniform foundation movements. The present study also highlights the significance of comprehensive geological integration and the critical role of multi-physics field monitoring systems in forecasting environmental impact and ensuring project integrity and safety.

## Methods

In this study, loading combinations of temperature drop, uplift pressure due to seepage at the limestone roof, and time dependent reservoir water load are incorporated in a large-scale finite element model for investigating the THM coupling response at the dam site (Supplementary Fig. S8).

We use a statistical method to obtain the target temperature drop of the rock strata. According to field measurements of pre-impounding ground temperatures and post-impounding seepage path, it is presumed that the temperature drop field follows a bilinear elliptical distribution, and an attenuation law is defined with respect to the distance to the center. Such a distribution introduces five parameters ***x*** for the field definition of temperature change (Supplementary Figs. S9, S10a). 6,048 sets of elliptical distribution parameters denoted as ***X*** were generated and each taken as inputs for a transient finite element calculation. Thus, a feedforward neural network based regression analysis is adopted to estimate the mapping relationship ***f*** relating temperature drop fields ***X ***to calculated valley deformations ***Y***. Finally, optimization analyses are carried out using the displacement data from field measurements as restraint conditions. An optimal temperature drop field with an average of temperature drop at 7–9 °C is determined. Considering the geometry of the elliptical thermal field, the aforementioned cooling volume of 80 million m^3^ can be obtained when the length is about 300–500 m along the river, which is basically consistent with the effective range observed at the plunge pool shown in Fig. 3c.

The uplift water pressure within the limestone stratum by the infiltration of reservoir water is considered as surface loads exerting on the limestone roof. Its spatial distribution is determined from an inverse analysis of four parameters Δ*H*_1_, Δ*H*_2_, *L*_1_, *L*_2_ based on vertical displacements measured at the dam and foundation galleries (Supplementary Fig. S10b). The optimal pressure rise of 1.8 MPa and 1.0 MPa for Δ*H*_1_ and Δ*H*_2_ respectively are obtained.

A visco-elasto-plastic constitutive model is chosen for simulating the stress–strain behavior of the site rock strata, employing the Drucker–Prager strength criterion with non-associated flow potential, and a power law creep model for representing long-term inelastic deformations mobilized within the interlayer shear zones by the change of site hydrogeological conditions.

## Supplementary information

Supplementary information.
