# Supplementary material for: Mechanism for large-scale canyon deformations due to filling of large reservoir of hydropower project
Source: Sci Rep. 2020 Jul 22;10:12155. doi: 10.1038/s41598-020-69167-9 (PMC7376047; doi:10.1038/s41598-020-69167-9)
Supplement: Supplementary file 1 — Supplementary information. [file 41598_2020_69167_MOESM1_ESM.pdf]

Supplementary Materials for

Mechanism for large-scale canyon deformations due to filling of large reservoir of hydropower project

Hui Jiang, Chu-Han Zhang*, Yuan-De Zhou, Jian-Wen Pan, Jin-Ting Wang, Ming-Xin Wu, & Qi-Xiang Fan

*Correspondence to: zch-dhh@tsinghua.edu.cn

This PDF file includes:

Field Data
Figs. S1 to S10

1. Supplementary field data

(1) <u>Canyon deformations</u>

Besides the seven monitoring lines VD01-VD07 in the first design stage, two more lines, VD08 in 2014 and consecutively VD09 in 2016, were arranged at the dam abutments at an elevation of 561m and 610 m, respectively. A summary of canyon contractions along the transverse river direction at the Xiluodu dam site is given in Fig. S1, showing spatially distributed time histories spanning the period 2013-2018 from upstream to downstream. It should be noted that VD08 line was placed in a drainage adit and extended into both abutments at a maximum depth of 150 m (VD08′). A uniform distribution pattern was observed at the measure points along the depth direction into the abutments, and the measurements from the two new monitoring lines are very close to the incremental readings from the other seven lines.

Based on the field data from geodetic datum network, including 58 working datum points, Fig. S2 shows the time histories of vertical displacements at the downstream (corresponding maximum values are denoted by circles in Fig. 1a). It can be observed that the datum points on the left bank generally manifest continuous uplift deformation following reservoir impounding, whilst an upward trend after initial subsidence during the first storage cycle is shown by the measuring points on the right bank.

Measurements of canyon deformations, including canyon contractions and uplift movements at both banks, tend to converge gradually after four impounding cycles.

(2) <u>Seepage flow observed in the plunge pool</u>

Totally 40 thermometers and 30 measuring weirs were arranged on the drainage galleries of the plunge pool to measure seepage flow rate and water temperatures. From the measurements, Fig. S4a shows the total seepage flow rate at the plunge pool, which reached up to 12 m^3/min and

stabilized at about 5-6 m^3/min after five impounding cycles. Meanwhile, a rise of seepage water temperature, up to 30-35 °C, was observed during the early filling stage Fig. S4b). These data demonstrate that the largest seepage flow rate and temperature change occurred in the first two cycles of reservoir impoundment, consistent with the mobilization of most significant canyon contractions in the time period.

(3) <u>Recent exploration observations</u>

In the project design stage, 78 boreholes were drilled deep into the basalt and limestone strata for exploring the geological conditions and ground temperatures. Through statistical analysis of these field data, Fig. S5 presents the variation of mean ground temperature with reference to the elevation. A well-fitted linear relationship is found and the trend line indicates a geothermal gradient of about 6 ℃/100 m.

For mechanism investigation of the canyon deformations, a supplementary drilling exploration was undertaken from November 2018, including 9 deep holes into the limestone aquifer and 8 holes into the basalt strata. The average of the bore-hole temperature data with elevation is shown as the red polyline in Fig. S5. It is shown that a significant temperature drop reaching 5-9 ℃ occurred below the elevation of 200 m where the limestone roof emerges. This is close to the calculated value of 7.5 ℃ (see Methods).

It is worthy to note that when some interlayer shear zones of basalt strata were drilled through, an abrupt temperature jump was monitored, indicating the upflow of hot water from buried limestone aquifers. Two typical instances are presented in Fig. S6, showing that there existed heat exchange within the rock mass around interlayer shear zones C3-C4 (Fig. S6a) and C4-C6 (Fig. S6b).

Fig. S7 shows the distribution of field measurements from 9 boreholes drilling into the limestone strata. High uplift water head (97-208 m) and a large quantity of seepage flow rate (0.42-0.76 m^3/min) were observed in most boreholes. The left and right insets present panoramas of two typical limestone boreholes, illustrating a well-developed karst feature.

(4) <u>Supplementary simulation results</u>

Through decomposition analysis of load factors including temperature drop, uplift pressure, reservoir water load as well as creep behaviors, we found that the cooling of bedrocks makes the most significant contribution to canyon contractions, accounting for almost 90%, whilst reservoir load and creep deformation of bedrock make minor effects, accounting for 8% and 2%, respectively. Uplift pressure can cause a slight increase in canyon spans (Fig. S3a).

In terms of vertical deformation, the seepage pressure mainly accounts for the uplift movements at the dam base and downstream valleys. Taking the foundation rock at an elevation of 347 m as an instance (Figs. S3b and S3c), the cumulative uplift at the left bank is 12 mm, amongst which the contributions of reservoir load, temperature drop, creep deformation and seepage pressure are -9 mm, -27 mm, -1 mm and 49 mm respectively.

2. Supplementary Figures

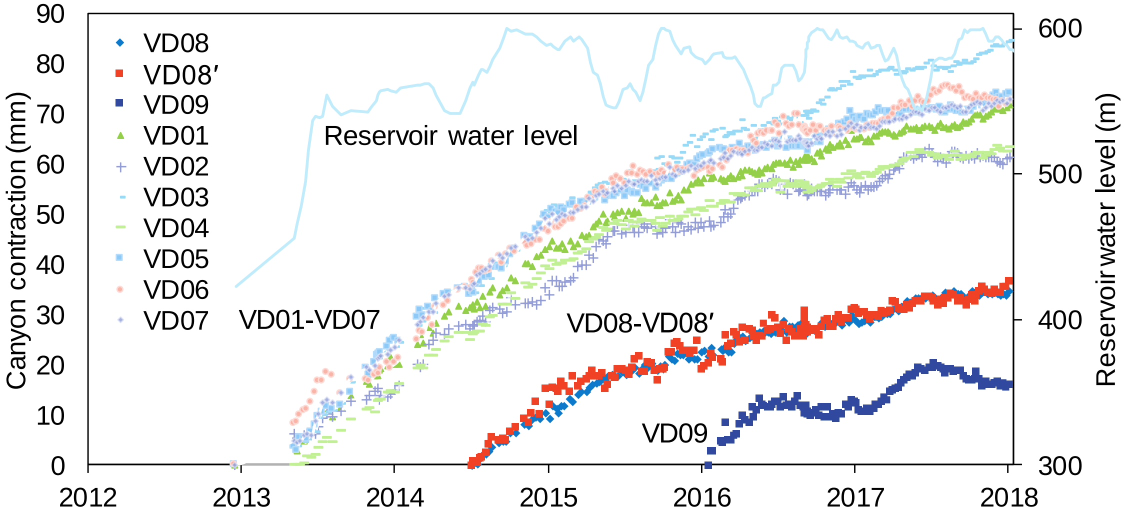

Fig. S1. Field measurement of canyon contractions (VD08 and VD09 were arranged at the dam abutment in 2014 and 2016, respectively. VD08′ was extended from VD08 at a maximum depth of 150 m into the tunnel).

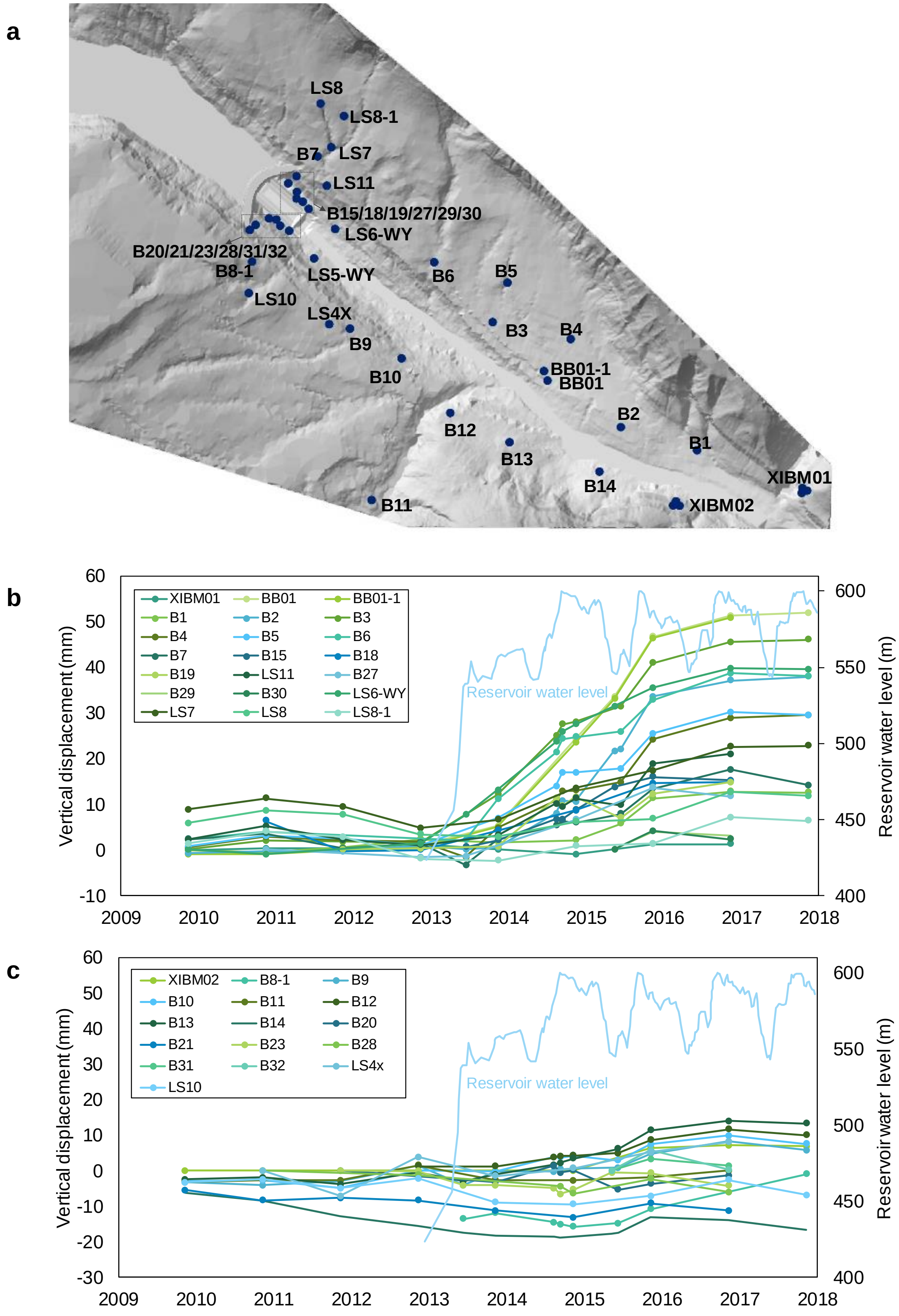

Fig. S2. Field measurement of vertical displacements on the downstream floor. (a) Working datum points; (b) Left bank; (c) Right bank.

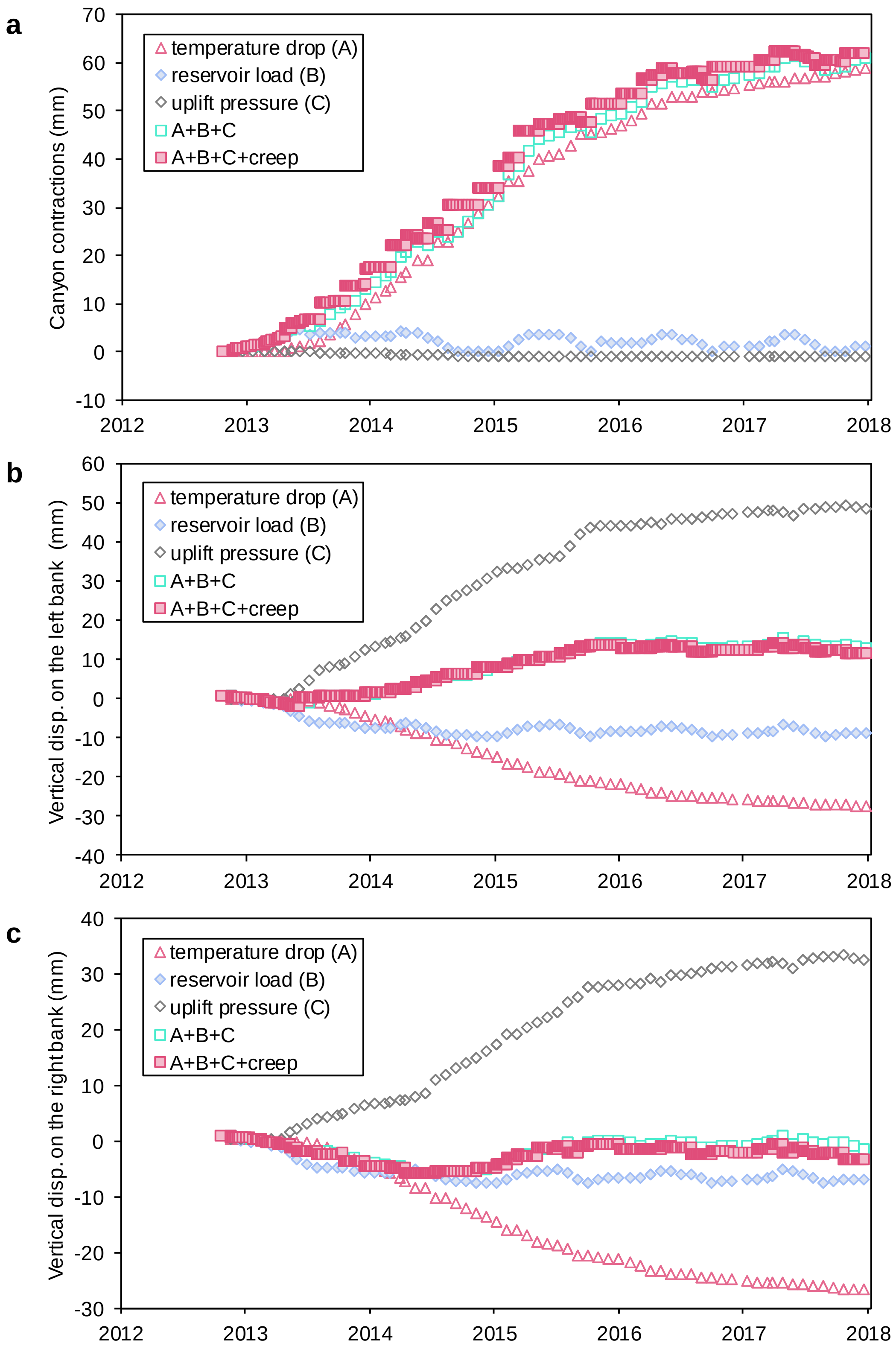

Fig. S3. Decomposition analysis of load factors. (a) Canyon contractions at the upstream with elevation of 610m; (b) Vertical displacements on the left dam base with elevation of 347m; (c) Vertical displacements on the right dam base at an elevation of 347m;

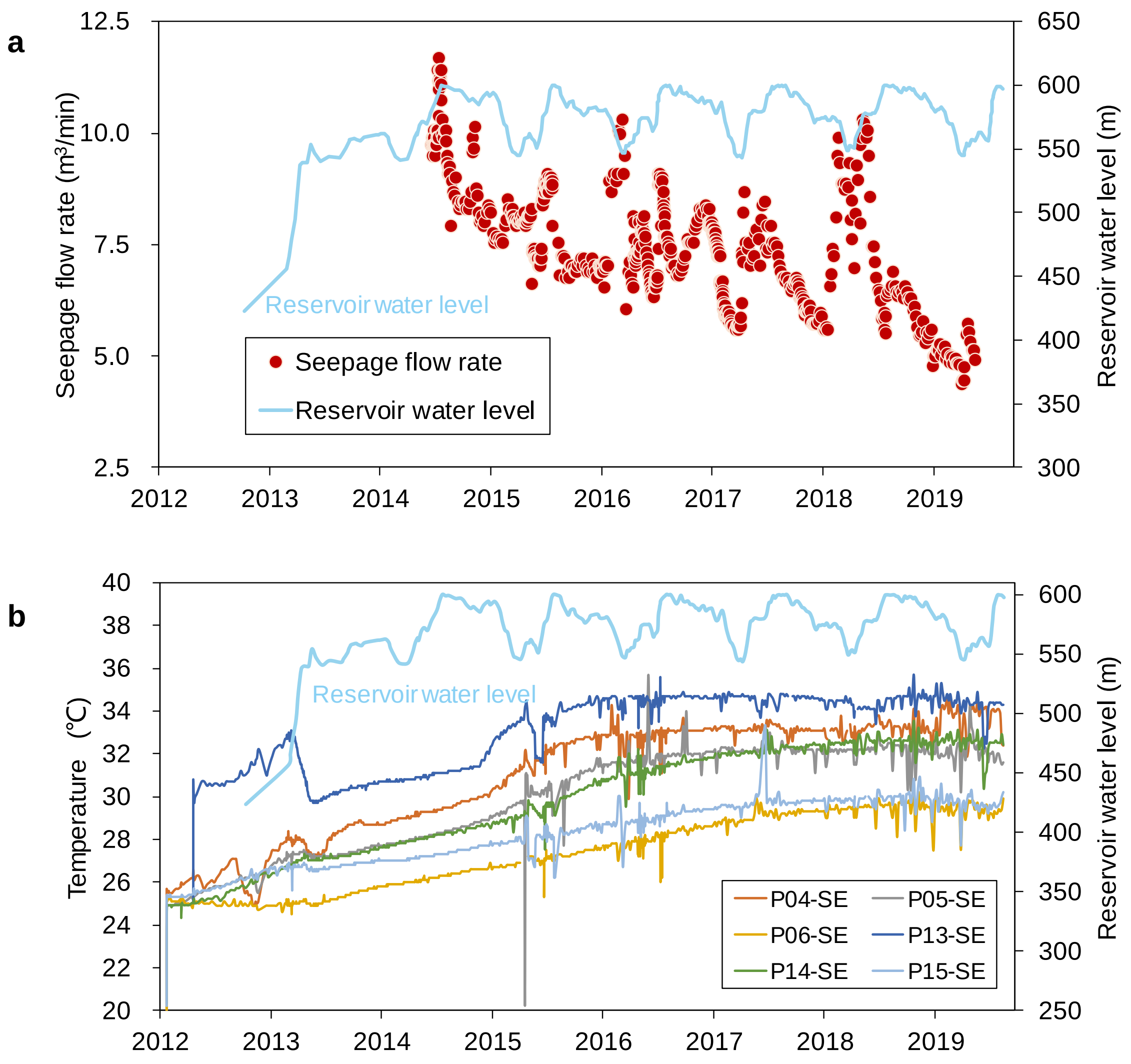

Fig. S4. Field measurements at the plunge pool downstream. (a) Seepage flow rate; (b) Seepage water temperature.

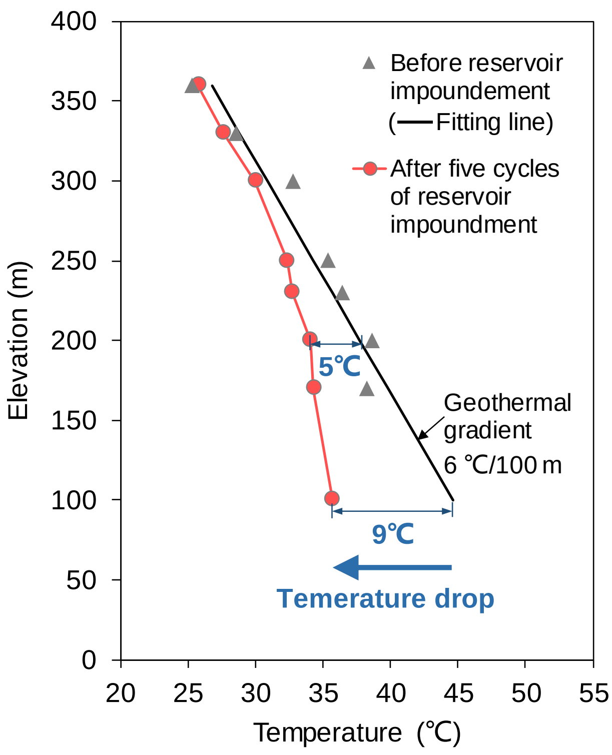

Fig. S5. Measured ground temperature drop after five cycles of reservoir impoundment

(Each point represents the average of measurements at the corresponding elevation).

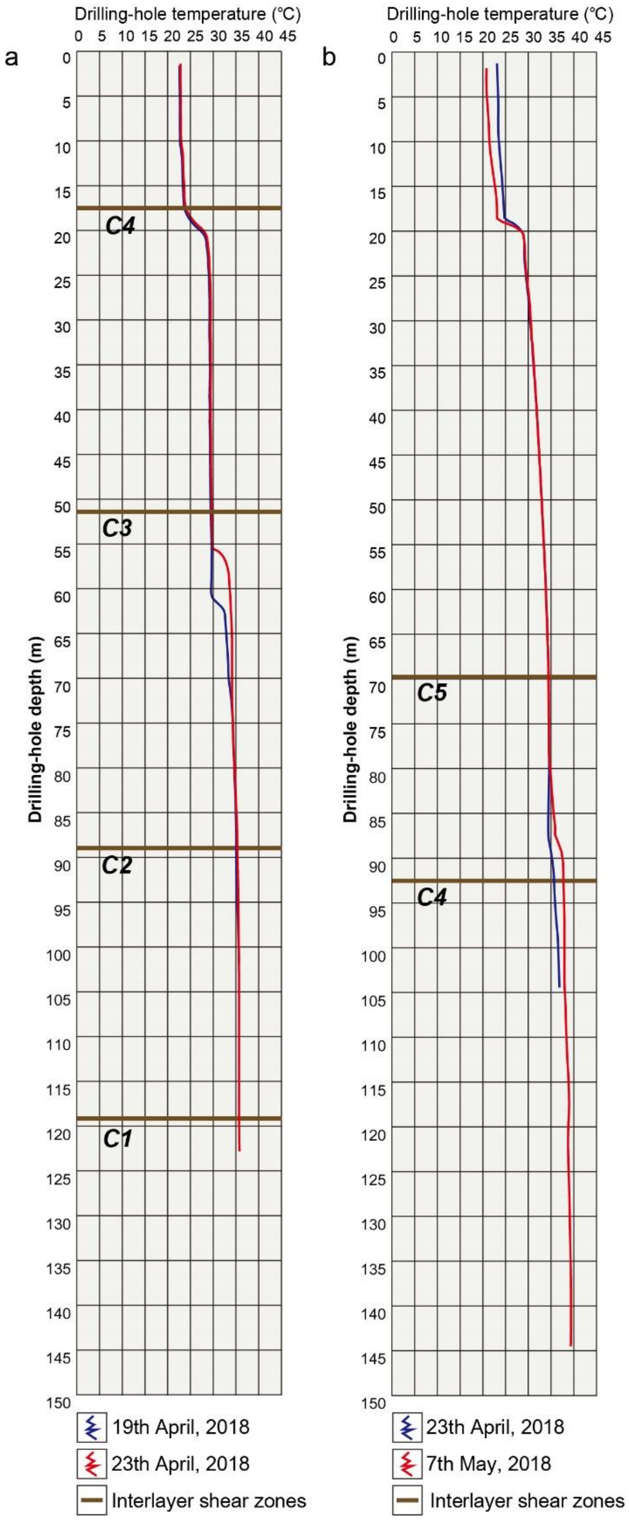

Fig. S6. Typical scenarios of temperatures measured along the depth of boreholes.

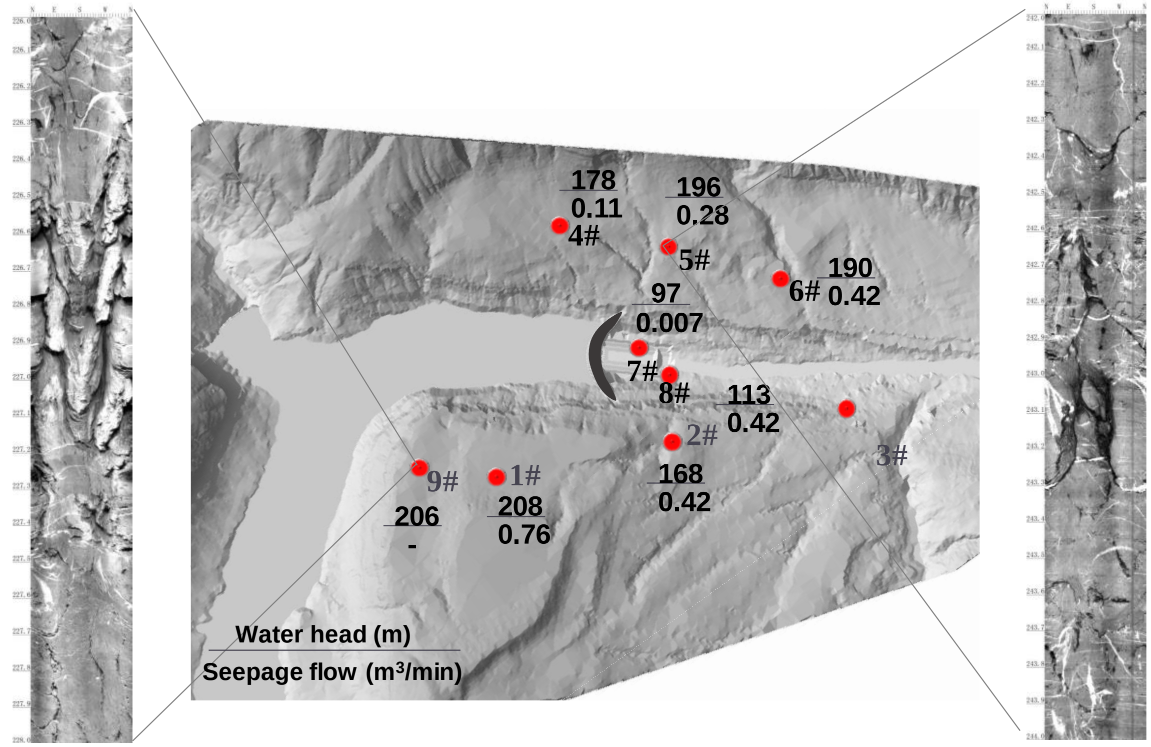

Fig. S7. Measurements of uplift water head and seepage flow rate from 9 boreholes into the limestone strata. The left and right insets present panoramas of limestone boreholes.

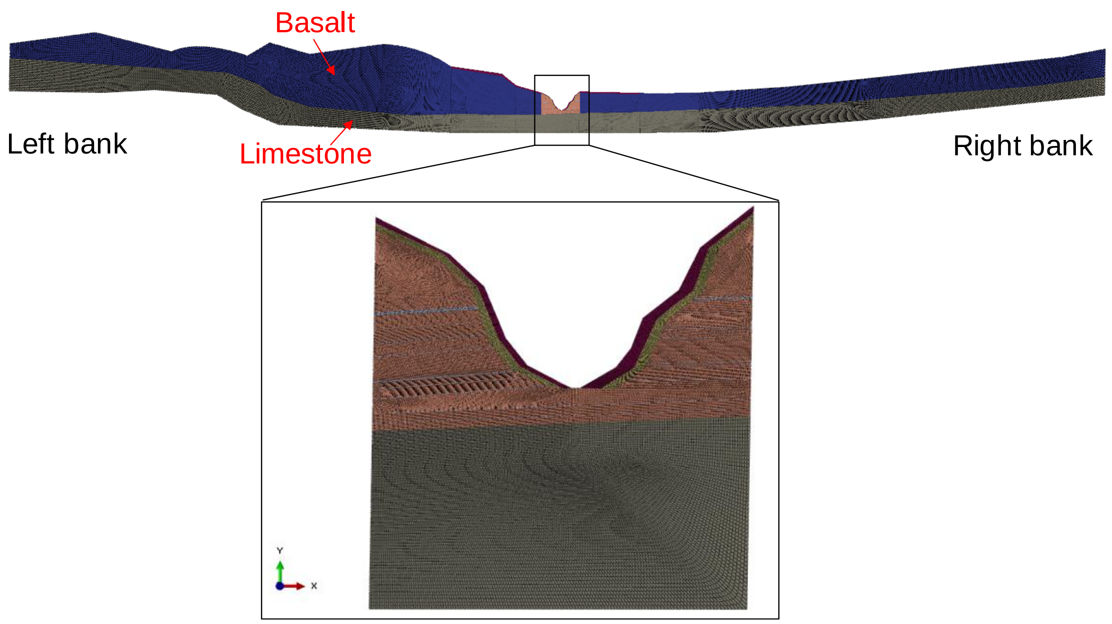

Fig. S8. Finite element mesh discretization.

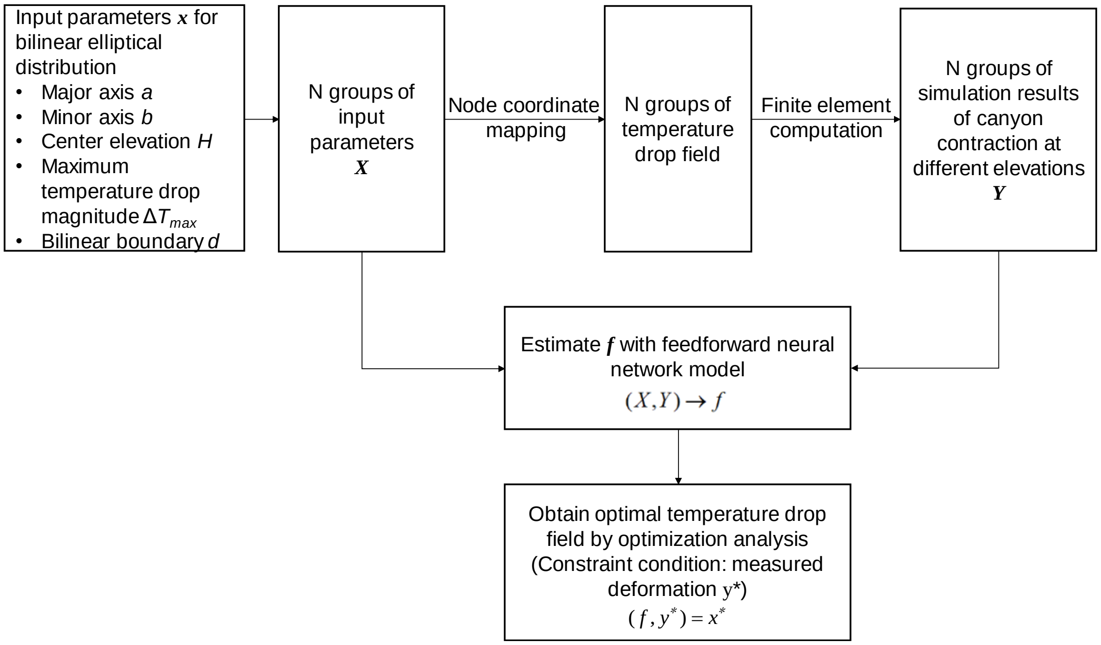

Fig. S9. Flow diagram for the feedforward neural network based regression model and optimization analysis.

a

b

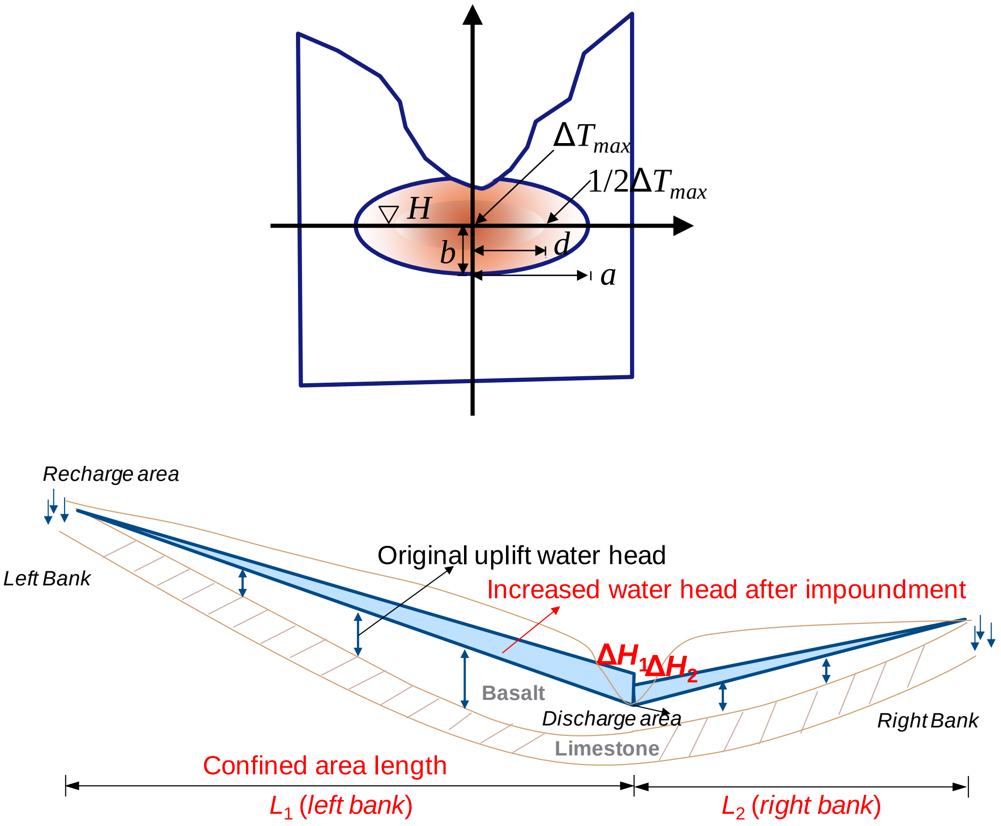

Fig. S10. Spatial distribution of cooling and uplift optimization model. (a) Temperature drop field model; (b) Uplift water pressure field model.
